# Arch height change during sit-to-stand: an alternative for the navicular drop test

**DOI:** 10.1186/1757-1146-1-3

**Published:** 2008-07-28

**Authors:** Thomas G McPoil, Mark W Cornwall, Lynn Medoff, Bill Vicenzino, Kelly Forsberg, Dana Hilz

**Affiliations:** 1 Gait Research Laboratory, Program in Physical Therapy, Northern Arizona University, Flagstaff, Arizona, USA; 2 Medoff Physical Therapy, Flagstaff, Arizona, USA; 3 Department of Physiotherapy, University of Queensland, St. Lucia, Brisbane, QLD 4072, Australia

## Abstract

**Background:**

A study was conducted to determine the reliability and validity of a new foot mobility assessment method that utilizes digital images to measure the change in dorsal arch height measured at 50% of the length of the foot during the Sit-to-Stand test.

**Methods:**

Two hundred – seventy five healthy participants participated in the study. The medial aspect of each foot was photographed with a digital camera while each participant stood with 50% body weight on each foot as well as in sitting for a non-weight bearing image. The dorsal arch height was measured at 50% of the total length of the foot on both weight bearing and non-weight bearing images to determine the change in dorsal arch height. The reliability and validity of the measurements were then determined.

**Results:**

The mean difference in dorsal arch height between non-weight bearing and weight bearing was 10 millimeters. The change in arch height during the Sit-to-Stand test was shown to have good to high levels of intra- and inter-reliability as well as validity using x-rays as the criterion measure.

**Conclusion:**

While the navicular drop test has been widely used as a clinical method to assess foot mobility, poor levels of inter-rater reliability have been reported. The results of the current study suggest that the change in dorsal arch height during the Sit-to-Stand test offers the clinician a reliable and valid alternative to the navicular drop test.

## Background

The navicular drop test (NDT) has been widely used as a clinical method to assess foot mobility. The NDT has also been associated with lower limb musculoskeletal injuries [1-3]. Brody was one of the first to describe the NDT and he noted that it was helpful in evaluating the amount of foot mobility, specifically pronation, in runners [4]. Brody stated that the NDT was performed with the patient standing on a firm surface with the navicular bone marked bilaterally. The patient's subtalar joint was first placed in neutral position using palpation and the height of the navicular bone from the floor was marked on an index card placed on the medial aspect of the foot. The patient was then asked to relax their feet and the resulting lower position of the navicular bone was also marked on the card. To determine the degree of navicular drop, Brody stated that the height of the navicular bone in subtalar joint neutral position is subtracted from the height of the navicular bone in relaxed standing posture. Brody further noted that a normal amount of navicular drop was approximately 10 mm and that a drop or change in navicular height of 15 mm or more was abnormal. While Brody indicated that the NDT was an office procedure that he used to assess the amount of foot pronation, he failed to provide any normative data to explain the navicular drop values he provided in his paper [4]. In addition, he did not indicate whether the NDT demonstrated high levels of intra-rater and inter-rater reliability.

Since Brody's initial description of the NDT, several authors have attempted to determine the reliability of the measurement as well as establish normative values in a healthy population. Studies have reported NDT values ranging from 6 to 9 mm with standard deviations of between 3.4 and 4.2 mm. The mean NDT value for these studies was 7.3 ± 3.8 mm [5-8].

Intra-rater reliability of the NDT, assessed using the intraclass correlation coefficient (ICC) has been reported to be between 0.61 and 0.79 [6-8]. A possible issue with these previous studies was that all examiners were inexperienced in performing the NDT. To investigate whether examiner experience influenced intra-rater reliability of the NDT, Evans et al assessed the reliability of the NDT in 30 adults using four different podiatric physicians who had previous experience performing the NDT [9]. The mean navicular drop was 7.2 mm with the range from 0 to 20 mm. Using intraclass correlation coefficients, the intra-rater reliability for the four raters ranged from 0.51 to 0.77 with the inter-rater reliability 0.46. In a more recent study, Shultz et al attempted to determine whether multiple raters with varying years of clinical experience could be trained to perform the NDT with acceptable reliability and precision [10]. Four raters had from one to six years of clinical experience and were trained by a single instructor with two years experience performing the NDT. Based on intraclass correlation coefficients, the intra-rater reliability ranged from 0.91 to 0.97 for the four raters.

Studies investigating inter-rater reliability have reported ICC values ranging from 0.46 and 0.83 [7-10]. One possible factor contributing to the moderate to poor levels of inter-rater reliability for the NDT could be the difficulty in consistently placing the subtalar joint in its neutral position using palpation [11-14].

While the results of previous investigations indicate that the NDT has high levels of intra-rater reliability, poor levels of inter-rater reliability and the lack of normative data from a large cohort of health individuals prevents its use in situations where numerous clinicians at different clinical sites are required make the measurements (e.g., multi-center outcome studies, multi-practitioner practices). The most prominent issues related to lower levels of inter-rater reliability would appear to be the identification of the navicular tuberosity bony landmark as well as the consistency of placing the subtalar joint in neutral position using palpation. In light of these issues, new methods that are developed to assess the mobility of the foot should not require the clinician to identify specific anatomical bony landmarks or to place the foot in precise positions.

Hoppenfeld has described what he termed a "test for rigid or supple feet" in which the clinician observed the patient's feet first in sitting and then in standing [15]. Hoppenfeld noted that if the medial longitudinal arch was absent in both sitting and standing, the patient had rigid feet. He further noted that if the medial longitudinal arch is present in sitting but absent when standing, the patient had supple feet [15]. While the "Sit-to-Stand" test was described by Hoppenfeld as an observational examination only, possibly the change in medial longitudinal arch posture, as measured using the change in dorsal arch height, could be quantified during the "Sit-to-Stand" test. The advantage of quantifying the "Sit-to-Stand" test is that the need to place the foot in subtalar joint neutral position or to identify the navicular tuberosity, which is necessary to perform the NDT, is not required. If acceptable levels of reliability and validity of the "Sit-to-Stand" test can be demonstrated, an alternative method for assessing foot mobility would be available for clinicians and researchers. Thus, the purpose of this study was to determine the reliability and validity of a new foot mobility assessment method that utilizes digital images to measure the change in dorsal arch height measured at 50% of the length of the foot during the Sit-to-Stand test.

## Methods

### Participant Characteristics

The right and left feet of 275 participants (155 women and 120 men) were assessed to establish a mean and standard deviation for a reference population of convenience. Participants were recruited from the Northern Arizona University population and the surrounding Flagstaff, Arizona community. All participants met the following inclusion criteria: 1) no history of congenital deformity in the lower extremity or foot; 2) no previous history of lower extremity or foot fractures; 3) no systemic diseases that could effect lower extremity or foot posture; and 4) no history of trauma or pain to either foot, lower extremity, or lumbosacral region at least 12 months prior to the start of the investigation. The mean age of the 275 participants was 26.3 ± 11.8 years with a range of 16 to 70 years. The mean age for the female and male participants was 23.9 ± 10.2 and 29.6 ± 13.1 years, respectively. The Institutional Review Board of Northern Arizona University (IRB # 04.0017) approved the protocol for data collection and all participants provided informed written consent prior to participation. Although no standardized "warm-up" protocol was used for the participants prior to data collection, each participant had been weight bearing and ambulating for at least 2 hours while conducting their normal activities of daily living.

### Procedures

**Figure 1:**
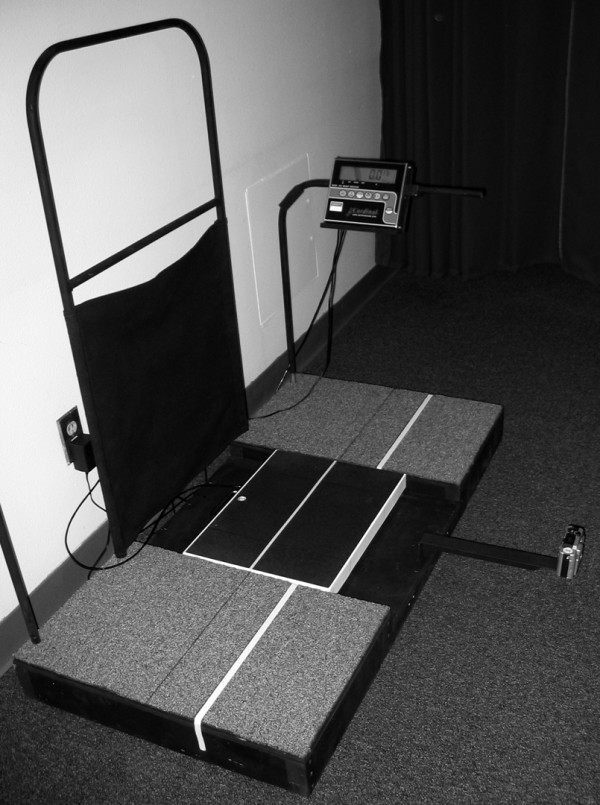
**Platform with weight scale used for digital image capture**.

**Figure 2:**
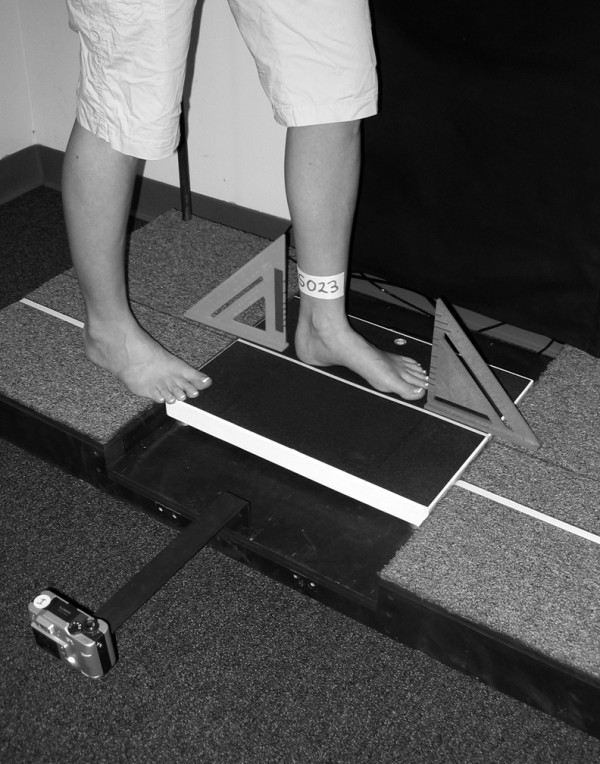
**Placement of the participant's left foot for the weight bearing image capture**.

**Figure 3:**
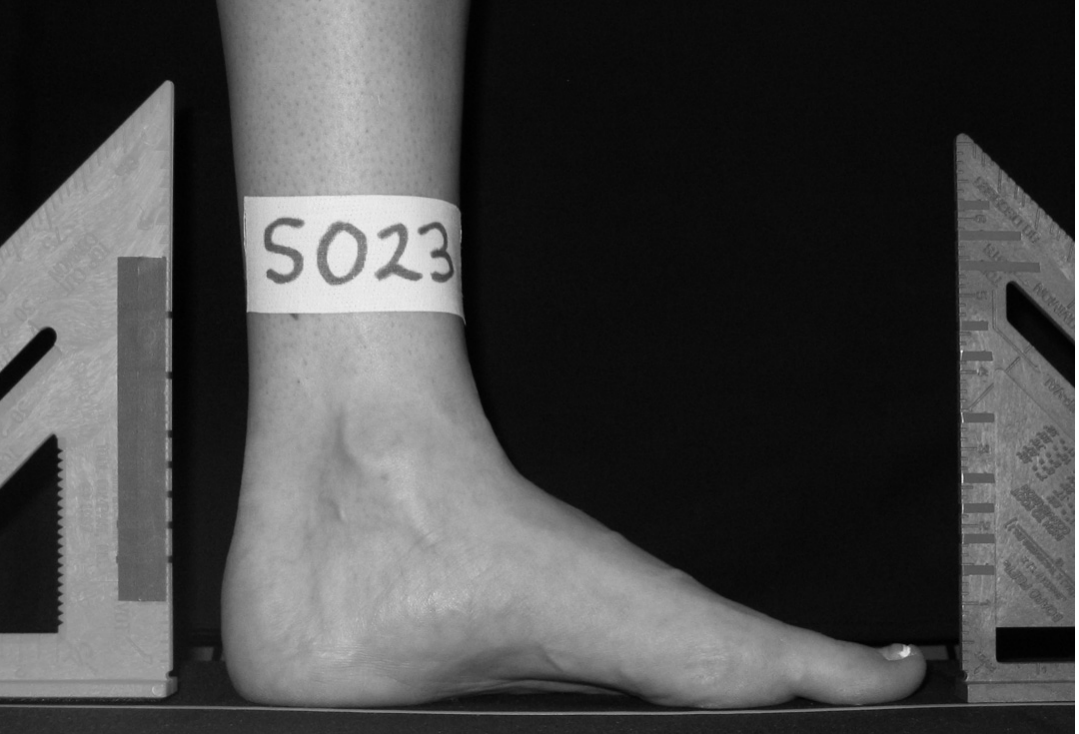
**Example of the 50% weight bearing digital image with known linear distances**.

Digital images were recorded for both feet while the participant stood placing 50% of their body weight on the foot being assessed as well as in non-weight bearing. A wood platform was constructed with a handrail for the participant to use to maintain balance as well as to ensure that the weight scale with digital read-out was level with the standing surface (Figure 1). For the 50% weight-bearing image of the left foot, the participant was asked to first place their left foot in the middle of a calibrated weight scale along a yellow line that divided the scale into equal halves (Figure 2). The participant was then instructed to place their right foot on a white line that was 15 cm away from the yellow line with the tip of the right big toe positioned at the end of the left heel. This ensured a clear digital image of the medial aspect of the left foot. Once positioned, the participant was asked to practice loading their left foot with 50% of their body weight while maintaining a relaxed foot posture. The participant was instructed to use the handrail for balance, relax their feet and to ensure equal loading on each extremity. Once the participant could place 50% of their body weight on their left foot while equally loading both extremities, relaxing the foot and maintaining their balance, the participant was instructed to position their left lower leg so that it was perpendicular to the supporting surface and a digital image of the medial aspect of the left foot was obtained (Figure 3). The tendons of the anterior compartment of the lower leg were palpated to verify that they were relaxed. Once the weight-bearing image for the left foot was obtained, the procedure was repeated for the right foot.

**Figure 4:**
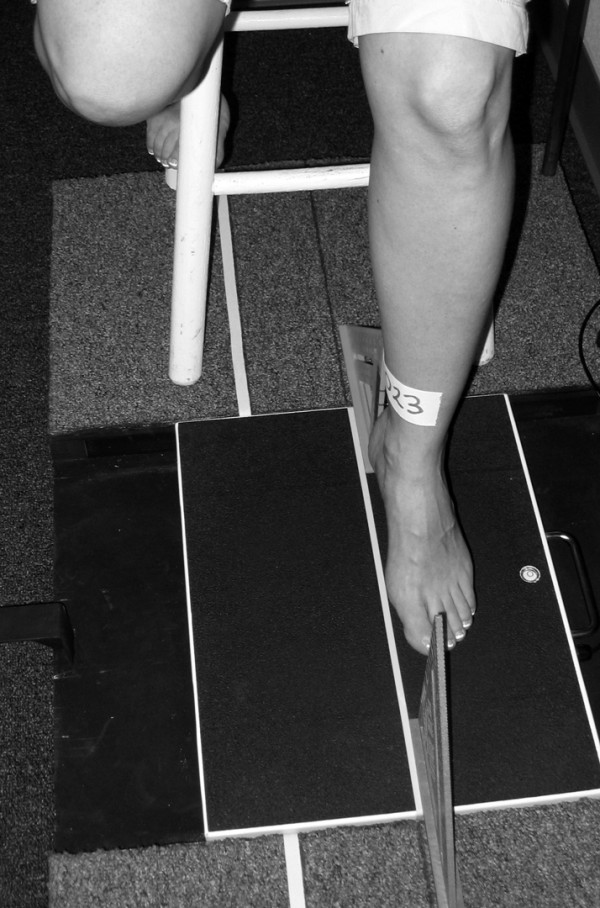
**Placement of participant's left foot for non-weight bearing image capture**.

**Figure 5:**
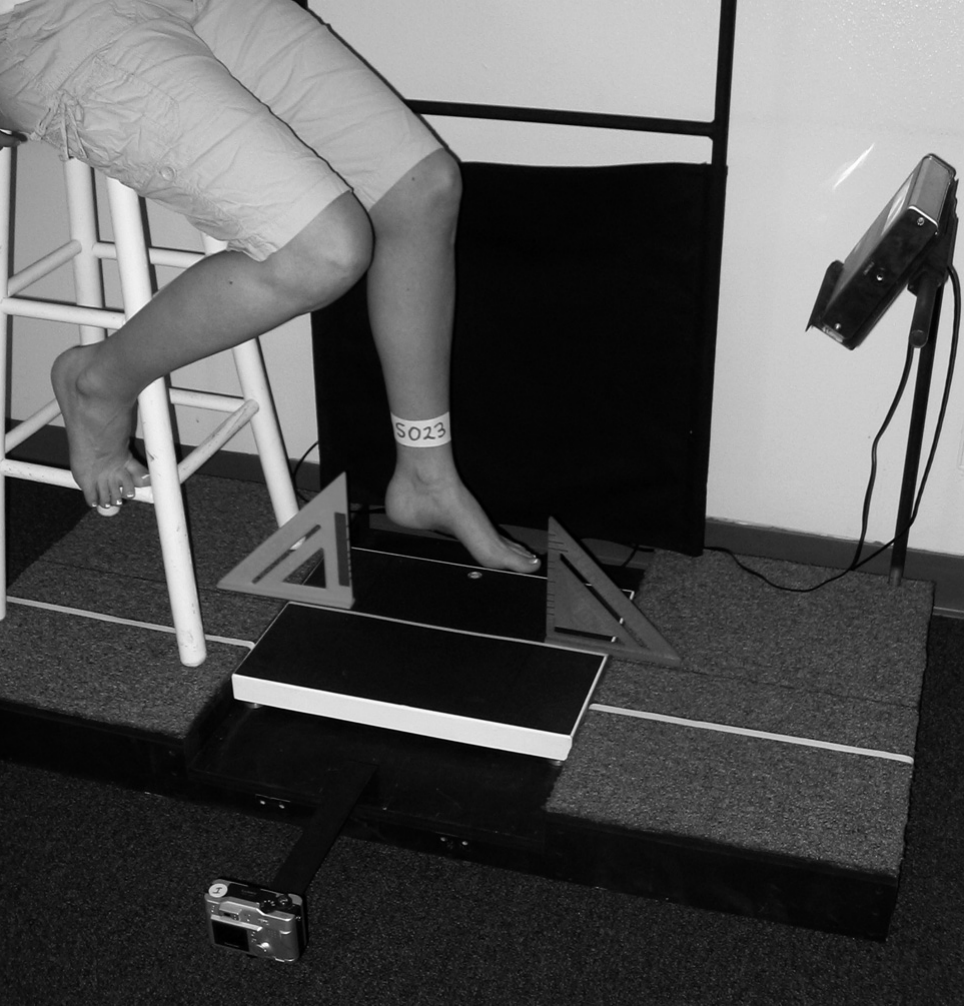
**Participant positioned on bar stool for the non-weight bearing image capture**.

**Figure 6:**
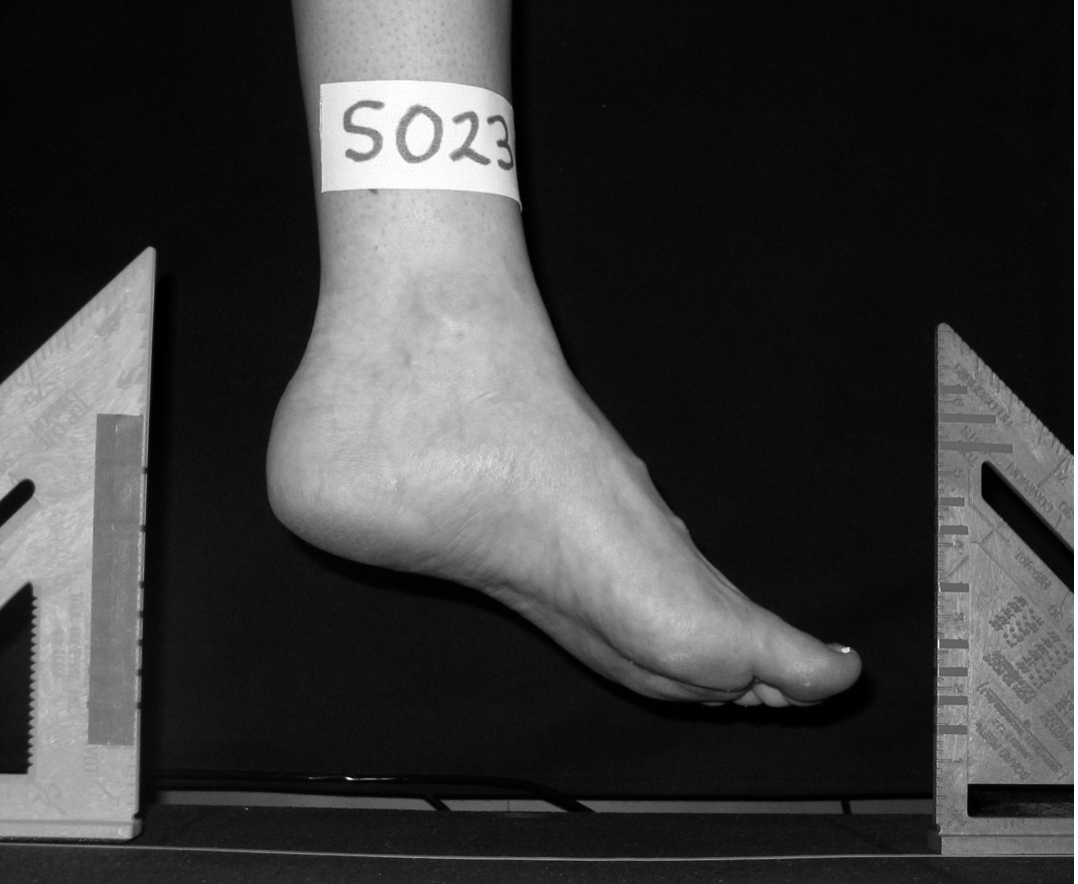
Example of the non-weight bearing digital image with known linear distances.

For the non-weight bearing image, the participant was asked to sit on a bar stool and place their left foot over the surface of the weight scale (Figure 4 and 5). To ensure consistency among participants as well as the position of the foot to the digital camera, the tips of the toes of the foot being photographed were positioned so that they were between 10 and 13 cm above the surface of the weight scale. Once the placement of the left foot above the weight scale was acceptable, the medial aspect of the left foot was visually aligned with the same white line used for the weight bearing foot image. When the left foot was properly positioned, the participant was instructed to relax their foot and a digital image of the medial aspect of the left foot in non-weight bearing was recorded (Figure 6). Again, the tendons of the anterior compartment of the lower leg were palpated to verify that they were relaxed. Once the non-weight bearing image for the left foot was obtained, the procedure was repeated for the right foot.

A digital camera (Model #DMC-LC20, Panasonic Corp., Secaucus, NJ 07094) was used to record all foot images. The camera was attached to a metal bar that was positioned 61 cm from the yellow line in the middle of the scale to ensure that the same focal length was used for all of the digital images (see Figure 1). Two objects of known distance were always included in the field of view of the digital camera to permit calibration of all measurements (see Figures 3 and 6).

All digital images obtained for both feet of each participant were downloaded onto a computer using Adobe Photoshop software (Adobe Photoshop version 7.0, Adobe Systems Inc., San Jose, CA 95110) and then printed using a color LaserJet printer (Model # 4600, Hewlett-Packard, Palo Alto, CA 94304). Each of the four images per participant was enhanced with Adobe Photoshop using the "Auto Color" feature. No other enhancements or modifications were done to any of the digital images. From the digital image, total foot length was measured using a ruler and was defined as the distance from the most posterior aspect of the heel to the tip of the hallux. For both the weight bearing and non-weight bearing image for each foot, the total foot length was first determined by measuring the distance from the most posterior aspect of the heel to the tip of the hallux. The total foot length was then divided in half to determine 50% of the total foot length. The dorsal arch height in weight bearing (ArchHtWB) was determined by measuring the vertical height from the supporting surface to the dorsum of the foot at 50% of the total foot length. To determine the dorsal arch height in non-weight bearing (ArchHtNWB), a reference line was first drawn from the most inferior point of the heel pad to the most inferior point of the first metatarsal head. From the reference line, a second line perpendicular to the reference line was drawn at 50% of the total foot length. The ArchHtNWB was then determined by measuring the distance from reference line to the dorsum of the foot along the perpendicular line. Each measurement was manually performed three times and the average was recorded. The ArchHtNWB measurement was subtracted from the ArchHtWB measurement to determine the change in arch height from Sit-to-Stand (ArchHtDIFF).

### Determination of Reliability and Validity

To establish intra-rater and inter-rater reliability for the measurements, two physical therapy students with no experience managing foot and ankle problems and one physical therapist with 12 years of experience managing foot and ankle problems were asked to assess the left and right foot images of 12 randomly selected participants (48 images). The 12 participants included 6 males and 6 females with a mean age of 23.9 ± 1.0 years. Each rater was given a set of written instructions on how to perform the measurements, but was not given any verbal instructions to permit the assessment of reliability to be more clinically applicable. Each rater was required to perform total foot length and dorsal arch height measurements for all 48 images twice with at least a one-week interval between the measurements. Each rater was blinded from any information that could be used to identify the participants they were assessing.

To establish validity, lateral radiographs were taken of the right foot of the same 12 participants used for the reliability assessment. Using the same foot placement protocol previously described, the participant stood on the same weight scale with the lateral border of their right foot against the radiographic cassette and placed 10%, 50%, and 90% of their body weight on the right foot. While Williams and McClay have previously reported the validity for the weight bearing dorsal arch height measurement, they only assessed radiographic images obtained while their participant's stood with 10% and 90% of body weight placed on foot [16]. Thus, it was decided to obtain radiographs on each foot assessed with 10%, 50%, and 90% body weight to justify the use of 50% body weight for this Sit-to-Stand technique. To permit comparison with the radiographs, a digital image of the medial aspect of the right foot of the 12 participants was obtained while they stood with 10%, 50%, and 90% of their body weight on the right foot. Once the three weight-bearing radiographs were completed, a non-weight bearing radiograph was obtained using the same foot placement protocol previously described. A wooden block was used to ensure proper placement of the radiographic cassette for the non-weight bearing x-ray. For all four radiographs, the x-ray unit was positioned vertical to the supporting surface and the center of the x-ray beam was placed just superior to the lateral malleolus. The distance from the x-ray tube to the foot was 101.6 cm and the exposure setting used was 150 mA at 54 kV. The same protocol for obtaining the four lateral radiographs was used for all 12 participants. To prevent possible magnification and parallax errors when obtaining the total foot length and dorsal arch height measurements from the lateral radiographs, two metal pieces of known length were placed on the top and at both ends of the x-ray film to serve as linear calibration references during data analysis. A fourth rater was used to obtain the total foot length and dorsal arch height measurement three times for each radiograph, using the same written instructions provided to the reliability raters. This additional rater was a physical therapist with over 20 years experience managing foot and ankle problems and was used to analyze the radiographs to prevent possible measurement bias. Validity was established using the average of the three values for each radiographic measurement compared with the mean measurement values obtained from the digital images on the same 12 participants.

### Statistical Analysis

Type (2,1) intraclass correlation coefficients (ICC) were calculated to determine the consistency of each rater to repeatedly perform the measurements both individually (intra-rater) and in comparison to the other raters (inter-rater) [17]. In addition to ICC values, the standard error of measurement (SEM)[18] and 95% limits of agreement (95LA) statistics were also calculated as another index of the reliability of the measurement [19]. The SEM is a number in the same units as that of the original measurements and represents the way a single score will vary if the foot length and dorsal arch heights were measured more than once [18]. The 95LA statistic provides an indication of the variability of the difference between any two measurements of foot length, arch height or change in arch height. The level of reliability for the ICC was classified using the characterizations reported by Landis and Koch [20]. These characterizations were: *slight*, if the correlation ranged from 0.00 to 0.21; *fair*, if the correlation ranged from 0.21 to 0.40; *moderate*, if the correlation ranged from 0.41 to 0.60; *substantial*, is the correlation ranged from 0.61 to 0.80; and *almost perfect*, if the correlation ranged from 0.81 to 1.00.

*T *tests were used to determine whether differences existed between the left and right feet for the foot length and dorsal height measurements. To assess the validity of the dorsal arch height measurements, Pearson product moment correlation coefficients were calculated to compare values from the digital images and those from the radiographs. An alpha level of .05 was established for all tests of statistical significance.

## Results

Average values for the dorsal arch height for both 50% weight bearing and non-weight bearing as well as the difference between the two measurements are shown in Table 1. The decrease in the arch height for all participants from non-weight bearing to 50% weight bearing was 1.00 cm. This change represented 13.4% of the arch height in non-weight bearing. This percentage of change was found to be 12.9% and 13.5% for males and females respectively. The intra-rater and inter-rater reliability values for all three raters are shown in Tables 2, 3, 4, and 5. Intra-rater reliability ICC values for total foot length, dorsal arch height and change in arch height for all raters ranged from 0.73 to 0.99 with SEM values ranging from 0.06 to 0.19 centimeters. The mean inter-rater measurement bias was -0.09 ± 0.21 cm. Inter-rater reliability ICC values for the same measurements ranged from 0.73 to 0.98 with SEM values ranging from 0.07 to 0.16 centimeters. The mean intra-rater measurement bias was 0.03 ± 0.23 cm.

**Table 1:** Descriptive statistics for foot length, arch height 50% WB, arch height non-WB, and ArchHtDIFF

	**Foot Length**		**Arch Height 50% WB**		**Arch Height Non-WB**		**Arch Height DIFF**	
	
	**Mean**	**SD**	**Mean**	**SD**	**Mean**	**SD**	**Mean**	**SD**
**All ** **Participants** ** (n = 550)**	24.87	2.05	6.49	0.61	7.49	0.64	1.00	0.36
**Females** ** (n = 310)**	23.73	1.47	6.20	0.49	7.17	0.52	0.97	0.36
**Males** ** (n = 220)**	26.36	1.71	6.90	0.50	7.91	0.53	1.02	0.34

**Note: **Mean and SD values in centimeters, SD = Standard Deviation, WB = Weight Bearing

**Table 2:** Intra-rater and inter-rater mean and standard error of the measurement (SEM).

	**Intra-rater**						**Inter-rater**	
	**Rater 1**		**Rater 2**		**Rater 3**			

	**Mean**	**SEM**	**Mean**	**SEM**	**Mean**	**SEM**	**Mean**	**SEM**

**Weight Bearing ** **Arch Height**	5.98	0.10	5.98	0.06	5.99	0.06	5.99	0.07
**Weight Bearing** ** Foot Length**	24.52	0.15	24.64	0.17	24.73	0.16	24.67	0.15
**Non-Weight** **Bearing Arch** ** Height**	7.12	0.08	7.30	0.10	7.39	0.11	7.28	0.11
**Non-Weight** **Bearing Foot** ** Length**	24.57	0.016	24.69	0.16	24.64	0.19	24.63	0.16
**AH Difference**	1.18	0.09	1.31	0.11	1.40	0.14	1.30	0.10

**Note: **Mean values in centimeters

**Table 3:** Intra-rater and inter-rater reliability coefficients (ICC).

	**Intra-rater**						**Inter-rater**	
	**Rater 1**		**Rater 2**		**Rater 3**			

	**ICC**	**95% CI**	**ICC**	**95% CI**	**ICC**	**95% CI**	**ICC**	**95% CI**

**Weight Bearing** ** Arch Height**	0.82	0.42 – 0.95	0.92	0.73 – 0.98	0.96	0.85 – 0.99	0.95	0.86 – 0.99
**Weight Bearing ** **Foot Length**	0.76	0.28 – 0.93	0.97	0.88 – 0.99	0.99	0.98 – 0.99	0.95	0.86 – 0.99
**Non-Weight ** **Bearing Arch** ** Height**	0.84	0.48 – 0.96	0.73	0.22 – 0.92	0.78	0.33 – 0.94	0.73	0.42 – 0.92
**Non-Weight** **Bearing Foot** ** Length**	0.98	0.94 – 0.99	0.99	0.94 – 0.99	0.99	0.94 – 0.99	0.98	0.95 – 0.99
**AH Difference**	0.92	0.78 – 0.98	0.88	0.71 – 0.97	0.93	0.81 – 0.98	0.92	0.78 – 0.98

**Note: **95% CI = 95% Confidence Interval

**Table 4:** Intra-rater bias, standard deviation and 95% limits of agreement.

	**RATER 1**			**RATER 2**			**RATER 3**		
	**BIAS**	**SD**	**95% LA**	**BIAS**	**SD**	**95% LA**	**BIAS**	**SD**	**95% LA**

**Weight Bearing** ** Arch Height**	-0.09	0.24	-0.57 – 0.39	0.02	0.17	-0.31 – 0.35	-0.08	0.13	-0.33 – 0.18
**Weight Bearing** ** Foot Length**	0.03	0.76	-1.46 – 1.52	-0.01	0.25	-0.51 – 0.48	-0.00	0.08	-0.16 – 0.16
**Non-Weight** ** Bearing Arch** ** Height**	0.07	0.20	-0.32 – 0.46	0.03	0.30	-0.56 – 0.62	0.21	0.33	-0.44 – 0.86
**Non-Weight** **Bearing Foot** ** Length**	-0.03	0.19	-0.40 – 0.34	-0.05	0.20	-0.43 – 0.33	0.11	0.20	-0.29 – 0.51
**AH Difference**	0.16	0.28	-0.38 – 0.70	0.01	0.37	-0.71 – 0.73	0.29	0.40	-0.49 – 1.07

**Note: **Values are in centimeters, SD = Standard Deviation, 95% LA = 95% Limits of Agreement

**Table 5:** Inter-rater bias, standard deviation and 95% limits of agreement.

	**Rater 1 vs Rater 2**			**Rater 1 vs Rater 3**			**Rater 2 vs Rater 3**		
	**BIAS**	**SD**	**95% LA**	**BIAS**	**SD**	**95% LA**	**BIAS**	**SD**	**95% LA**

**Weight Bearing** ** Arch Height**	0.00	0.11	-0.21 – 0.21	-0.01	0.16	-0.32 – 0.30	-0.01	0.14	-0.19 – 0.26
**Weight Bearing** ** Foot Length**	-0.12	0.41	-0.92 – 0.68	-0.21	0.37	-0.93 – 0.51	-0.09	0.11	-0.31–0.13
**Non-Weight** **Bearing Arch** ** Height**	-0.18	0.29	-0.76 – 0.40	-0.27	0.33	-0.92 – 0.38	-0.09	0.24	-0.56 – 0.38
**Non-Weight ** **Bearing Foot** ** Length**	-0.12	0.17	-0.45 – 0.21	-0.07	0.26	-0.58 – 0.44	0.05	0.16	-0.26 – 0.36
**AH Difference**	-0.14	0.29	-0.70 – 0.43	-0.23	0.28	-0.77 – 0.31	-0.09	0.25	-0.59 – 0.41

**Note: **Values are in centimeters, SD = Standard Deviation, 95% LA = 95% Limits of Agreement

The results of the *t *tests indicated that there were no significant differences (p > .05) between the left and right feet for any of the foot measurements assessed. The results of the Pearson correlations between the digital image measurements and the radiographic measurement showed that the radiographic measurements were all positively correlated with the digital image measurements. These correlation values were 0.91 at 10% WB, 0.93 at 50% WB, 0.89 at 90% WB, 0.92 for non-weight bearing, and 0.12 for the difference between NWB and 50% WB. The results of the 95LA statistical analysis between the digital and radiographic measurements are contained in Table 6. This analysis showed that the radiographic measurements were between 0.47 and 1.43 cm less than that of the digital image measurements. Although there was a consistent bias for the radiographic measurements to be less than the digital image, the standard deviation of these differences was relatively small.

**Table 6:** Bias and 95% limits of agreement between the radiographic and digital image measurements.

	**Bias (cm)**	**SD (cm)**	**95% Limits Of Agreement**
**10% WB**	-0.65	0.21	-1.06 to -0.25 cm
**50% WB**	-0.97	0.19	-1.34 to -0.59 cm
**90% WB**	-0.88	0.22	-1.32 to -0.44 cm
**Non-WB**	-1.43	0.26	-1.94 to -0.93 cm
**AH Difference From** ** Non-WB To 50% WB**	-0.47	0.36	-1.18 to 0.24 cm

**Note**: WB = weight bearing

## Discussion

The purpose of this study was to determine the reliability and validity of a new foot mobility assessment method, the ArchHtDIFF, which utilizes digital images obtained during the Sit-to-Stand test. While the NDT has been widely used as a clinical method to assess foot mobility, previous studies have shown that the NDT has poor levels of inter-rater reliability. This prevents the NDT from being used as a measurement tool in multi-center outcome studies where numerous clinicians at different clinical sites are required to collect data. The key issues that can lead to lower levels of inter-rater reliability with the NDT are the identification of the navicular tuberosity bony landmark as well as the consistency of placing the subtalar joint in neutral position using palpation.

Since the use of digital images to quantify the change in dorsal arch height during the Sit-to-Stand test does not require the identification of bony landmarks or the palpation of a specific foot posture, the authors hoped this would lead to higher levels of measurement reliability among multiple raters. Franettovich et al have shown that the reliability of dorsal arch height measures have higher levels of reliability with the participant standing with 50% of their body weight on each foot compared with the 10% and 90% weight bearing condition [21]. While Williams and McClay established validity for the 10% and 90% weight bearing conditions, the validation of the 50% body weight has not been established [16]. Thus, it was also important for the authors of the current study to establish the validity for using the 50% weight bearing condition.

For foot length in 50% weight bearing, the intra-rater reliability ranged from 0.76 to 0.99 for all three raters and the inter-rater reliability was 0.95. For dorsal arch height in 50% weight bearing, the intra-rater reliability for ranged from 0.82 to 0.96 for the three raters with the inter-rater reliability being 0.95. For non-weight bearing foot length, the intra-rater reliability ranged from 0.98 to 0.99 for the three raters and the intra-rater reliability was 0.98. For non-weight bearing dorsal arch height, the intra-rater reliability ranged from 0.73 to 0.84 for the three raters and the intra-rater reliability 0.73. Using the ICC classification scheme described by Landis and Koch, the ICC values for both intra-rater and inter-rater would be classified as substantial to almost perfect [20]. The intra-rater and inter-rater SEM values were all less that 5% of the mean measurement value. In addition, the 95LA analysis showed that both the differences between two measurements of a single rater or between two raters were relatively small. In light of the results of the ICC, 95LA, and in particular the SEM analyses, the authors believed that the measurements were consistent (Tables 4 and 5). Rater one frequently had lower reliability compared to the other two raters, but this finding was not consistent for all variables measured and the 95LA for rater one is generally comparable to that of the other two raters. As such, there does not appear to be a clear effect of rater experience upon the reliability of taking these measurements.

While Williams and McClay did not assess non-weight bearing dorsal arch height, they reported inter-rater reliability ICC values of 0.79 for 10% weight bearing and 0.77 for 90% weight bearing [16]. In the current study using digital images, the inter-rater ICC value was 0.95 for ArchHtWB and 0.73 for ArchHtNWB. Based on the ICC, SEM and 95LA values obtained, the authors believe that the intra-rater and inter-rater consistency to assess the change in dorsal arch height during Sit-to-Stand was acceptable. It should be noted, however, that participants were not measured on two or more occasions by each rater. As such, the effect of participant positioning between measurements was not assessed. Although this could have caused intra-rater reliability to be higher than what might occur in a clinical setting, the authors feel that its effect was minimal since inter-rater reliability was found to be very high and in those situations, participants did change positions for each rater.

The same variables from both the digital images as well as lateral radiographs were used to assess the validity of the dorsal arch height change during the Sit-to-Stand test. The lowest correlation for arch height was noted between the digital image and the radiograph for 90% weight bearing (r = 0.89). The correlation for arch height between the digital image and the radiograph for both 10% and 50% weight bearing was 0.91 and 0.93, respectively. The correlation between the digital image and the radiograph for non-weight bearing dorsal arch height was 0.92. Since the correlations for digital images obtained during 10% and 50% weight bearing explained over 85% of the arch height measured from the radiograph, the authors believed that a high level of validity existed for the measurement of the dorsal arch height in 50% weight bearing. This is further supported by the 95LA analysis, which showed that 95% of the differences between the measurements were less than 1.02 cm. The correlation between the differences in arch height as measured by the two methods was low (r = 0.12), but the standard deviation of the bias between the two measurements was still relatively low (0.36 cm). As such, 95% of the differences were within 1.41 cm. Although the measurements from the digital image and the radiograph are considered to be reliable, they are not identical as shown by the bias between the two measurements. The radiographic measurement was between 0.65 and 1.43 cm less than that measured from the digital image. This discrepancy is most likely due to the effect of soft tissue in the digital image measurement. Clinicians and other using either method should be aware of this systematic difference between them. It should also be noted that only a single experienced clinician was used to obtain the measurements from the radiographs. As such, additional research should be conducted to better determine how well the results of this study could be generalized.

The mean difference between non-weight bearing and 50% body weight arch height for the 275 *right *feet was 1.01 ± 0.37 cm. The mean difference between non-weight bearing and 50% body weight arch height for the 275 *left *feet was 0.97 ± 0.34 cm. The values for both the right and left feet were found to be normally distributed (p < .01) based on the D'Agostino and Pearson Omnibus test [22]. Since the results of the t-test indicated no significant difference between the left and right feet for the change in arch height between non-weight bearing and 50% body weight, the left and right foot data were pooled for further analysis.

The mean difference between non-weight bearing and 50% body weight arch height for all 550 feet was 1.00 ± 0.36 cm. Again, these values were found to be normally distributed (p < .01). For the purpose of using the difference between non-weight bearing and 50% body weight arch height for classifying foot mobility, we suggest using a classification scheme previously described by McPoil and Cornwall based on mean and standard deviation values from the pooled data of 550 feet [23]. A foot would be classified as having normal foot mobility if the difference between non-weight bearing and 50% body weight arch height was within ± 1 standard deviation of the mean. A foot would be classified as having *increased mobility *if the difference between non-weight bearing and 50% body weight arch height was greater than 1 standard deviation from the mean. To be classified as having *decreased mobility*, the difference between non-weight bearing and 50% body weight arch height would be less than one standard deviation from the mean. Based on this classification scheme, a foot would be classified as having *increased mobility *if the difference between non-weight bearing and 50% body weight arch height was greater than 1.35 cm. If the difference between non-weight bearing and 50% body weight arch height were less than 0.64 cm, the foot would be classified as having *decreased mobility*. Using these classification criteria on the combined participant pool of 550 feet, 396 would be classified as having normal foot mobility, 83 would have increased foot mobility, and 71 would have decreased foot mobility.

Brody stated that when using the NDT a normal amount of navicular drop was approximately 10 mm and that a drop or change in navicular height of 15 mm or more was abnormal [4]. While Brody failed to provide any normative data to explain the navicular drop values he provided in his paper, in the current study the mean ArchHtDIFF was 1.0 cm and based on one standard deviation from the mean, 1.35 cm would be considered as indicative of increased mobility.

In addition to providing information regarding foot mobility, the ArchHtWB value also appears to be predictive of foot posture during mid-stance in walking and mid-support in jogging. Franettovich et al has previously reported that the ArchHtWB measured in 50% weight bearing explained 66% of the variance associated with arch height measured at mid-stance in walking and 83% of the variance in arch height measured at mid-support in jogging [21]. Thus, using ArchHtWB provides the clinician with information regarding the posture of the foot during dynamic activities, such as walking and jogging, while the ArchHtDIFF provides an index of foot mobility.

A limitation in the proposed new method used to assess ArchHtDIFF is that the digital images must be downloaded from the camera, slightly enhanced using commercially available software, and then printed so that measurements can be obtained. While the ArchHtDIFF provides a method of assessment that has acceptable levels of reliability and validity, the method used to obtain the measurements may be too time consuming for the clinician. Future research should focus on developing a method to obtain the ArchHtDIFF that can be done easily and quickly in the clinic.

Another limitation of this study is that it was conducted entirely on asymptomatic individuals. As such, the normal values reported in this study may or may not be representative of individuals who have had an injury or who have some type of systemic disease such as rheumatoid arthritis.

## Conclusion

The findings of this study demonstrate that the difference in the dorsal arch height in non-weight bearing and the dorsal arch height in 50% weight bearing, as measured using the Sit-to-Stand test, provides the clinician with a reliable and valid alternative to quantify foot mobility in comparison to the navicular drop test. In addition, normative data on a large group of healthy participants is provided. While the method described for obtaining the ArchHtDIFF does require the clinician to process the digital images for the necessary measurements, based on the results of this study future research can focus on developing a less time-intensive method for measuring the ArchHtDIFF in the clinic.

## Competing interests

The authors declare that they have no competing interests.

## Authors' contributions

TGM conceived the study, participated in the design of the study, and carried out data analyses. MWC conceived the study, participated in the design of the study, and carried out data analyses. LM coordinated and carried out data analyses. BV participated in the design of the study and carried out data analyses. KKF coordinated and carried out data analyses. DH coordinated and carried out data analyses.
