# Arch height change during sit-to-stand: an alternative for the navicular drop test

**DOI:** 10.1186/1757-1146-2-17

**Published:** 2009-05-18

**Authors:** Thomas G McPoil, Mark W Cornwall, Lynn Medoff, Bill Vicenzino, Kelly K Fosberg, Dana Hilz

**Affiliations:** 1 Gait Research Laboratory, Program in Physical Therapy, Northern Arizona University, Flagstaff, Arizona, USA; 2 Medoff Physical Therapy, Flagstaff, Arizona, USA; 3 Department of Physiotherapy, University of Queensland, St. Lucia, Brisbane, QLD 4072, Australia

## Abstract

Correction to McPoil TG, Cornwall MW, Medoff L, Vicenzion B, Fosberg K, Hilz D. Arch height change during sit-to-stand: an alternative for the navicular drop test. Journal of Foot and Ankle Research 2008; 1:3.

## Correction

Correction to be made on the spelling of an author's name and email address in the publication of this work [1], as the mistake was not caught prior to publication. The name to be corrected is Kelly K. Fosberg and the correct corresponding email address is kellykaydpt@yahoo.com.
